# A process to foster pathology-related effects of design primes – how orthopedic patients might benefit from design features that influence health behaviour intention

**DOI:** 10.3389/fpsyg.2023.1211563

**Published:** 2023-11-20

**Authors:** Jonas Rehn-Groenendijk, Kai Schuster, Helena Müller, Evangelia Chrysikou

**Affiliations:** ^1^Darmstadt University of Applied Sciences, Darmstadt, Germany; ^2^Bartlett Faculty of the Built Environment, University College London, London, England, United Kingdom

**Keywords:** design primes, health behaviour change, material priming, evidence-based design, design methodology

## Abstract

A growing body of literature mainly in the context of consumer research indicates that the formal-aesthetic and conceptual design of objects can influence users’ thoughts, emotions and even behavioural patterns. While there is strong evidence regarding these effects on actual purchasing decisions, evidence on the effect of aesthetic design features (e.g., haptics, colour) on health-related mental concepts and intentions for health behaviour change is scarce. Based on insights from material and conceptual priming, this article illustrates the research-driven and evidence-based design process of two design primes and comprises pre-tests and an experiment in two settings on the effect of design on health behaviour focusing i.a. on intention for health behaviour change. In an evidence-based and research-driven process, two lecterns were designed to work as primes, i.e., to have a positive vs. negative influence on several mental constructs (sense of control, sense of coherence, resiliency, self-efficacy) and health-related intention. The lecterns differed mainly in terms of aesthetic appearance (e.g., material, colour, proportion, steadiness). They were tested in (a.) a university setting with students (*n* = 83) and (b.) a clinical setting with orthopaedic rehabilitation patients (*n* = 38). Participants were asked to perform an unrelated task (evaluation of an unrelated product) while standing at and using the lecterns. Overall, t-tests and Mann–Whitney-U tests show no significant differences but differing tendencies in a mentioning task. When asked to name health-promoting activities, in the clinical setting, participants using the “positive” prime (i.e., the steady lectern, *n* = 13) mentioned more sport-related aspects on average and a higher portion of sport-related aspects of their answers than participants using the “negative” prime (*n* = 11). In the university setting (positive: *n* = 36; negative *n* = 38), no such differences emerged. This finding gives reason to believe that the prime might be specifically effective in the clinical setting as it relates to physical activity being the most relevant topic of the patients’ pathology.

## 1 Introduction and theoretical background

An extensive body of literature supports the basic assumption of priming theory that the perception of a primary stimulus (prime) can influence the evaluation or behaviour regarding a subsequent stimulus (target) (e.g., Bargh and Chartrand, 2014; Kidder et al., 2018). The dominant explanation of this effect is based on the S*preading-Activation Theory* (Collins and Loftus, 1975) and the *Associative-Semantic Network Model* (Bower, 1981). According to Bower, “each distinct emotion such as joy, depression, or fear has a specific node or unit in memory that collects together many other aspects of the emotion that are connected to it by associative pointers “(p. 135). When a person is exposed to a prime (e.g., a word, picture or object), this stimulus can activate a knot in this semantic network. Spreading activation theory suggests that “when a concept is primed, activation tags are spread by tracing an expanding set of links in the network out to some unspecified depth” (Collins and Loftus, 1975, p. 409). While earlier studies primarily focused on the effect of words as stimuli, since the past decade, a growing body of research focuses on the effects of mere aesthetics such as colour (Mehta and Zhu, 2009), haptic sensations (Ackerman et al., 2010), smell (Lee and Schwarz, 2012) and other physical attributes (Lobel, 2014). In environmental psychology, action conditionality by spatial settings has been demonstrated using *Behaviour Setting Theory* (Barker, 1968). Barker (1968) observed that behaviour is shaped not only by individual factors, but by the specific space and its defined offers for action (*cf.* affordances according to Gibson, 1977). As a rule, there is even an alignment of action patterns, which he called *behaviour settings*. A specific feature of behaviour settings is therefore that differential characteristics of the persons present seem to be largely negligible and rather the spatial-material milieu is related to action. More recently, based on grounded cognition (Barsalou, 2008) and the notion of embodiment (Barsalou et al., 2003), bodily sensations and even the users’ posture when interacting with products or the built environment is receiving more attention (Van Rompay and Ludden, 2015). While these effects have been used intensely in the retail sector (e.g., Mikunda, 2008; Lindstrom, 2010, 2011, 2014), their potential is hardly acknowledged in therapeutic contexts. Some scholars have elaborated on explorative concepts and approaches for this purpose (Lockton et al., 2010; Laschke et al., 2014; Hassenzahl and Laschke, 2015) and others have addressed these potentials beyond the scope of mere physical interventions (e.g., Michie et al., 2014). However, there is hardly any design theoretical concept or conceptual framework that embraces aesthetic design features as a means to affect health behaviour or even health-related behavioural intentions. Elaborating on Schwarzers *Health Action Process Approach* (2004), Rehn (2018) has developed a design model that aims at connecting subtle design features to health behavioural effects. This model can be seen as a conceptual basis for the effect being discussed in this paper as it specifically outlines the mechanisms through which design primes and other subtle stimuli (e.g., Rehn, 2017) might affect health behaviour change. In this context, we refer to the term health behaviour in accordance with Kasl and Cobb’s (1966) early definition as “any activity undertaken by a person believing himself to be healthy, for the purpose of preventing disease or detecting it in an asymptomatic stage” (p. 531).

### 1.1 Design features as pathology-related material primes

**Figure 1:**
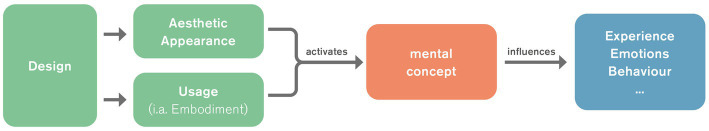
Process model of design primes.

Based on the aforementioned theoretical concepts, we define design primes as any formal aesthetic design feature that activates mental concepts that in turn influence situational experiences, emotions or behaviours (a.) by its mere aesthetic appearance (“style”) or (b.) by interacting with it (e.g., through principles of embodiment) (see Figure 1). In the retail sector this is commonly used for instance when fruits and vegetable are presented in wooden boxes instead of the plastics boxes they were actually transported in, to prime concepts of small-scale farms and nature as opposed to industrial farming. In paediatric hospitals, one might refer to the colourful and playful design features commonly used as design primes, since they aim at activating mental concepts of joy and playfulness as opposed to medical procedures.

While, as presented above, this concept has been implemented for decades (e.g., Packard, 1957), this paper illustrates approaches of utilising design primes in the context of health-promoting design. Therapeutic built environments such as hospitals and rehabilitation clinics are usually filled with aesthetic features that communicate to some extent a specific medical regime (Rehn-Groenendijk 2022). Whereas certain elements such as, e.g., flooring material or lighting are still mainly designed based on specific technical or functional needs (e.g., hygiene, safety) and an overall aesthetic concept, their additional subconscious effects on parameters such as clinical outcomes and health behaviour is often underestimated. Although prominent examples of the effect of design (Hernandez-Garcia et al., 2021) and in particular of aesthetic features on bio markers such as blood pressure are well established (e.g., “*white coat hypertension,”* Pickering, 1996), their potential use to support therapeutic processes is rarely embraced. At the same time, there is an ongoing discourse on the effect of the built environment related to by constructs and paradigms such as psychosocially-supportive design (Ulrich, 1991), healing environments (Dijkstra, 2009), evidence-based design (Malkin, 2008; Stichler and Hamilton, 2008; Halawa et al., 2020) and approaches linked to salutogenesis (e.g., Dilani, 2008). This can serve as a strong conceptual foundation to make use of these aesthetic features as subtle tools for promoting health. The multiple pathways through which these mechanisms might be effective are for instance illustrated by Salovey et al.’s (2000) work on the interrelation between emotional states and physical health and have been further elaborated by the work done in fields such as psychoneuroimmunology (e.g., Moraes et al., 2018).

We therefore propose, that formal-aesthetic design can not only contribute to technical goals (e.g., ergonomics) as well as affective responses (e.g., aesthetic appeal) but can directly address more complex mental concepts that in turn affect a number of subsequent thought patterns, emotions and behaviours. This applies in particular to the emotionally charged context of healthcare settings. In some medical fields, insights from this line of research might be difficult to implement and require small-scale interventions (e.g., Rehn-Groenendijk 2022). With regards to the specific field of orthopaedic rehabilitation, an overarching theme can be identified and addressed by design. In the German health system, which is the context of the experimental study described here, orthopaedic rehabilitation comprises a variety of pathologies. Most prevalent are postsurgical care after hip and knee joint replacement and treatments of rheumatic diseases. The hallmark of orthopaedic medicine can be seen in its focus on movement and physical activity. While most patients suffer from illnesses or pathologies that to some extent compromise physical activity (e.g., range of motion), one of the most profound improvements after therapeutic measures such as hip replacement is the almost immediate enhancement of movement ability. In line with this biomechanics approach are core perspectives of orthopaedic rehabilitation (e.g., Kulig and Burnfield, 2008). As a result, patients in orthopaedic rehabilitation can be expected to be highly sensitised towards physical activity. At the same time, sufficient motivation, commitment and health behavioural changes (e.g., nutrition and physical activity) usually are key components to achieve lasting positive health outcomes. In line with this, orthopaedic rehabilitation takes usually 3–6 weeks and comprises, in addition to specific medical treatments, educative elements to facilitate health behaviour change (for an overview see Ip, 2007). With regards to this, the built environment can have a significant complementary effect on both physiological and mental processes that can support therapeutic processes and outcomes (e.g., Ulrich et al., 2008; Ziegler, 2015; Ryan and Browning, 2020).

## 2 Methods a: development of design primes

According to the previous considerations, we assume that design and healthcare settings can benefit from applying principles of priming research. As illustrated above, the built environment (spaces as well as interior artefacts) to some extent exerts an effect on people in these environments. Hence, patients, staff and carers are constantly confronted and therefore influenced by formal-aesthetic features of their surrounding – whether they are purposely designed to do so or not. Understanding and making use of these effects in a health-promoting way poses broad potential for the healthcare system and is therefore worth investigating.

In order to do so, we developed two lecterns as exploratory design primes (negative vs. positive) in an iterative research-driven design process and tested their effects on individuals in two randomised controlled trials (university setting vs. clinical setting) (see also Rehn, 2019, p. 327–523; Rehn and Schuster, 2021).

The following research questions were addressed:

- *Do design primes that deliberately address constructs such as self-efficacy, autonomy, sense of control and valorisation have an effect on users’ self-efficacy, sense of control and resiliency while using them?*
- *Do these design primes associatively activate health behavioural mental concepts?*
- *Does the effect of these design primes depend on the relation between activated mental concept and individual pathology?*

In order to test design features as material and conceptual primes for health behaviour, an iterative research-driven (O'Grady and O'Grady, 2017) and evidence-based design (Hamilton, 2003) process was performed that aimed at addressing mental concepts such as self-efficacy, sense of control and health behavioural intention by design (see also Rehn and Schuster, 2021).

From a methodological point of view, the product category “lectern” was chosen as the functional dimension of the design primes, as they were neutral yet functional enough to be easily tested in both the university and the clinical setting. This was mainly due to the fact that a sham product test was used as a pretence to temporally use the lecterns for facilitating this testing narrative. In this way, individuals were asked to evaluate an unrelated medical product (pulse oximeter) while standing at the lectern and writing their review on the lectern table top. In line with this story, users’ attention would not be drawn to the otherwise uncommon object but rather give the impression of a standard equipment commonly used in product user tests. This allowed for major design adaptations without an aesthetic reference or baseline by users, i.e., whereas users have a mental model of how a table or chair looks and feels like, these lecterns were less likely to be related to prior expectations or knowledge. Therefore, the iterative design process focused on specific formal-aesthetic design features of the lectern to subtly address the above mentioned mental constructs (sense of control, sense of coherence, resiliency, self-efficacy) and health-related intention.

### 2.1 Mood boards

**Figure 2:**
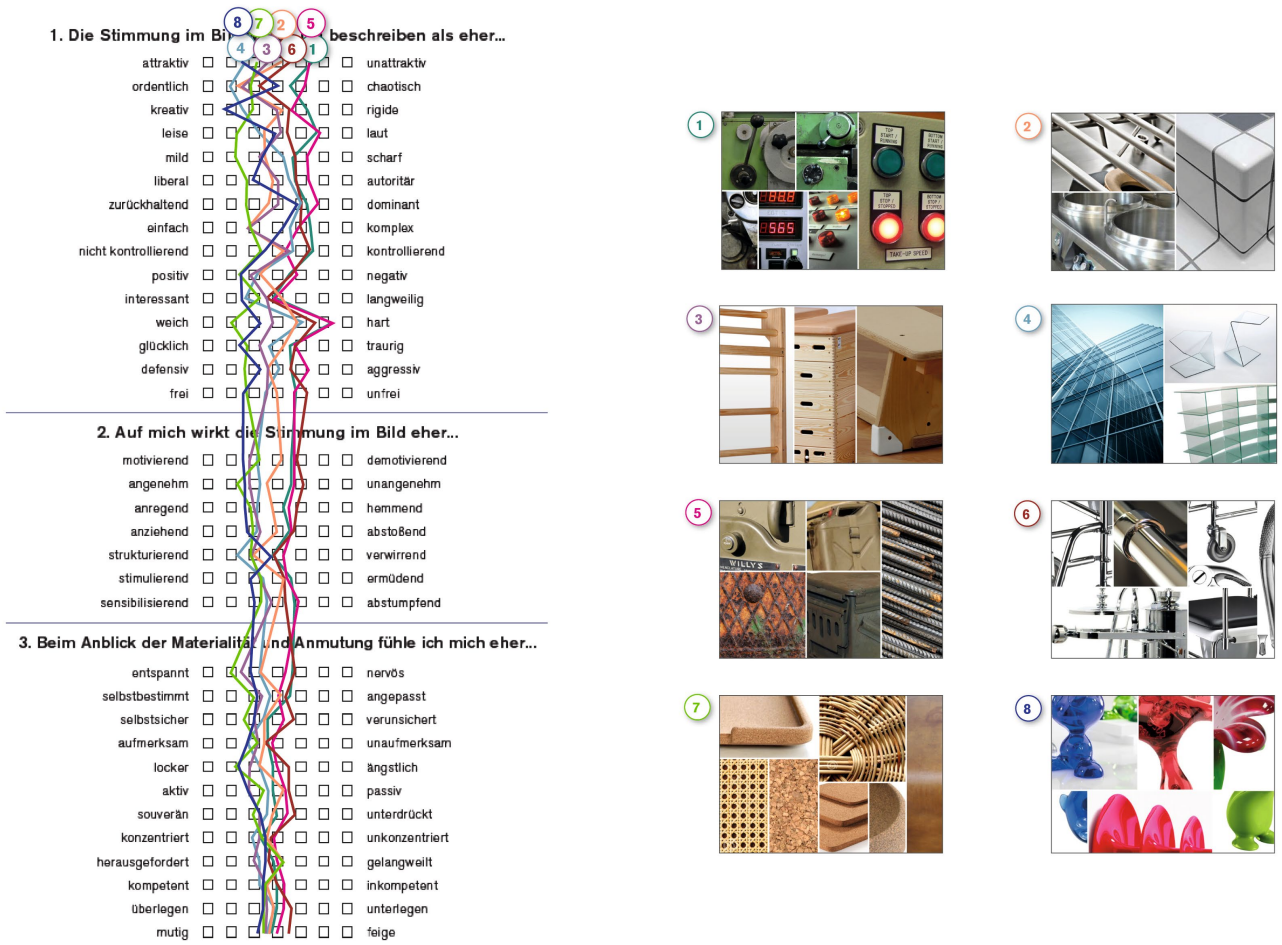
Mood boards and semantic profile.

Starting point of the design process was the creation of eight different mood boards representing formal aesthetic styles such as “playful” or “natural” (Figure 2). These boards were presented to 125 students, who evaluated the boards using semantic differentiation (Osgood, Charles 1979), a common practise to evaluate design variations (e.g., Li, 2022). The semantic differential was based on 34 items clustered around the seven categories “performance accomplishment,” “emotional arousal,” “sense of control,” “self-regulation,” “creativity,” “attractiveness,” “competence” and “therapeutic.” This structure and selection of items were inspired by the *User Experience Questionnaire* (Laugwitz et al., 2008) but adapted to fit the overall research question of this project.

Based on this survey, three mood boards (3, 7 and 8) could be allocated to be labeled as “positive” (having higher scores in terms of the aboved mentioned categories), whereas three other boards (1, 5 and 6) were scored markedly lower and could thus be labeled as “negative.” Two boards were evaluated ambiguously and were therefore excluded from the further process.

### 2.2 Initial ideation phase

In addition to these six mood boards, a number of explorative design concepts and ideas were created in an iterative design process using sketches, CAD tools and structured creativity workshops (Rehn, 2019, p. 379). In this workshop, four participants without specific prior knowledge on this topic were instructed to come up with associations, specific ideas and eventually design concepts around stimulus words such as “self care.” Usual creativity techniques (e.g., bisociation, brainstorming) were applied using sticky notes, markers and paper (*cf.* Braem, 1989; Noack, 2008). The ideas derived from the workshop were consolidated with prior concepts developed by author 1 and elaborated into explorative designs.

### 2.3 Research-driven design process for design primes

**Figure 3:**
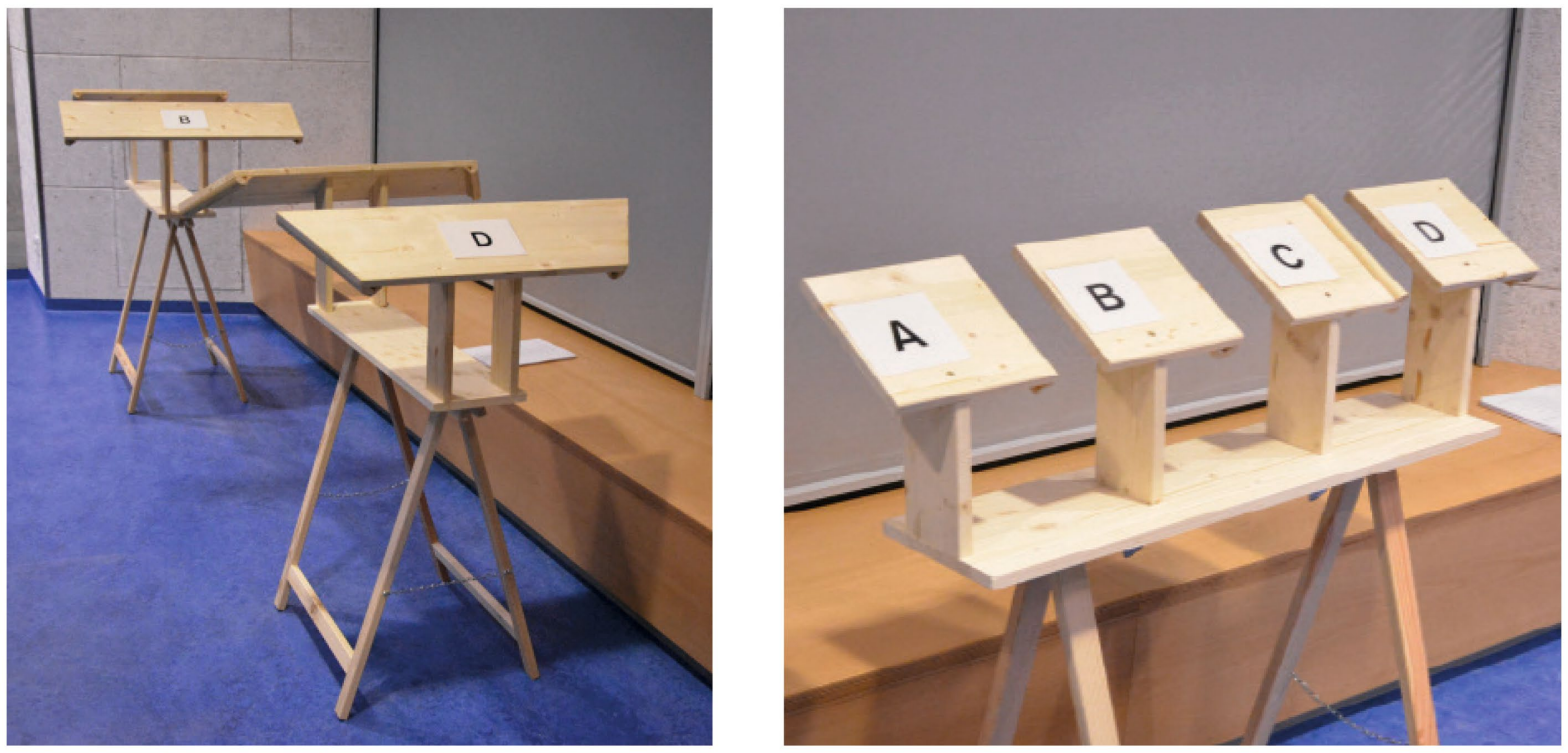
Illustration of pretest on design features.

**Figure 4:**
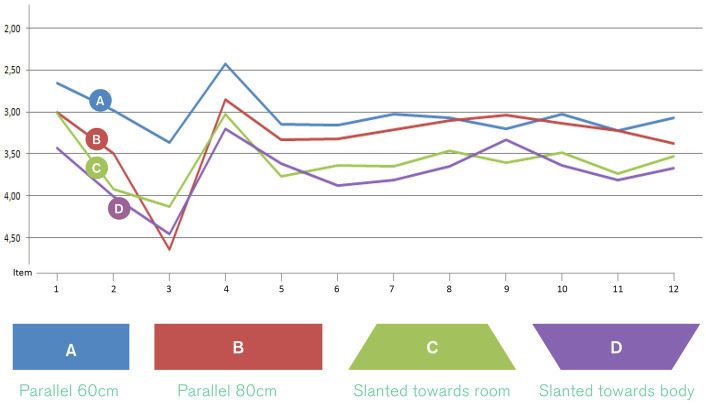
Design feature “basic shape” of lectern tabletop.

**Figure 5:**
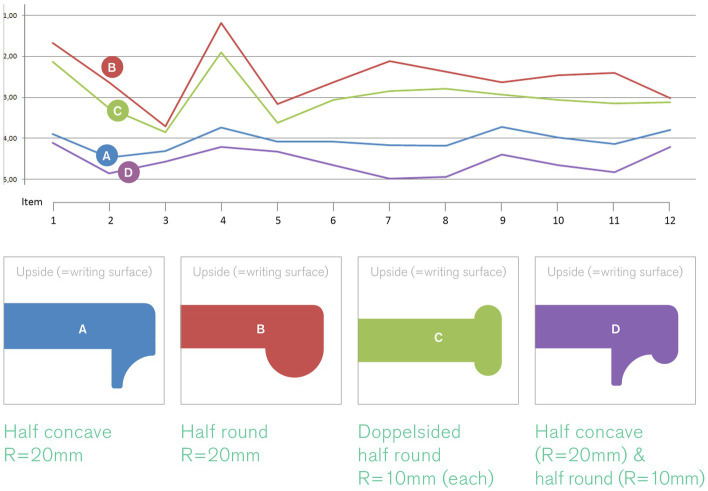
Design feature “handle shape.”

**Figure 6:**
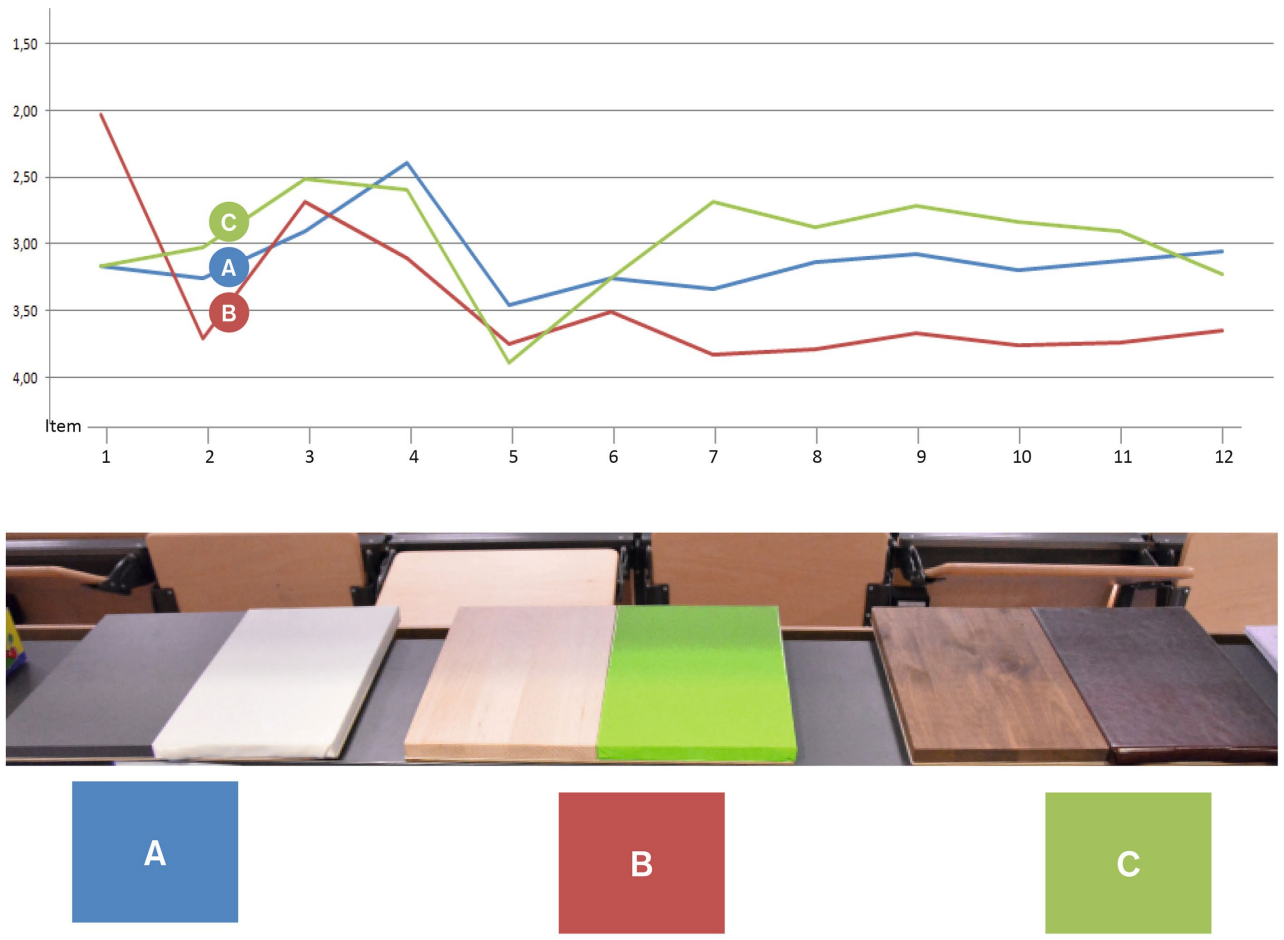
Design feature “material and colour.”

**Figure 7:**
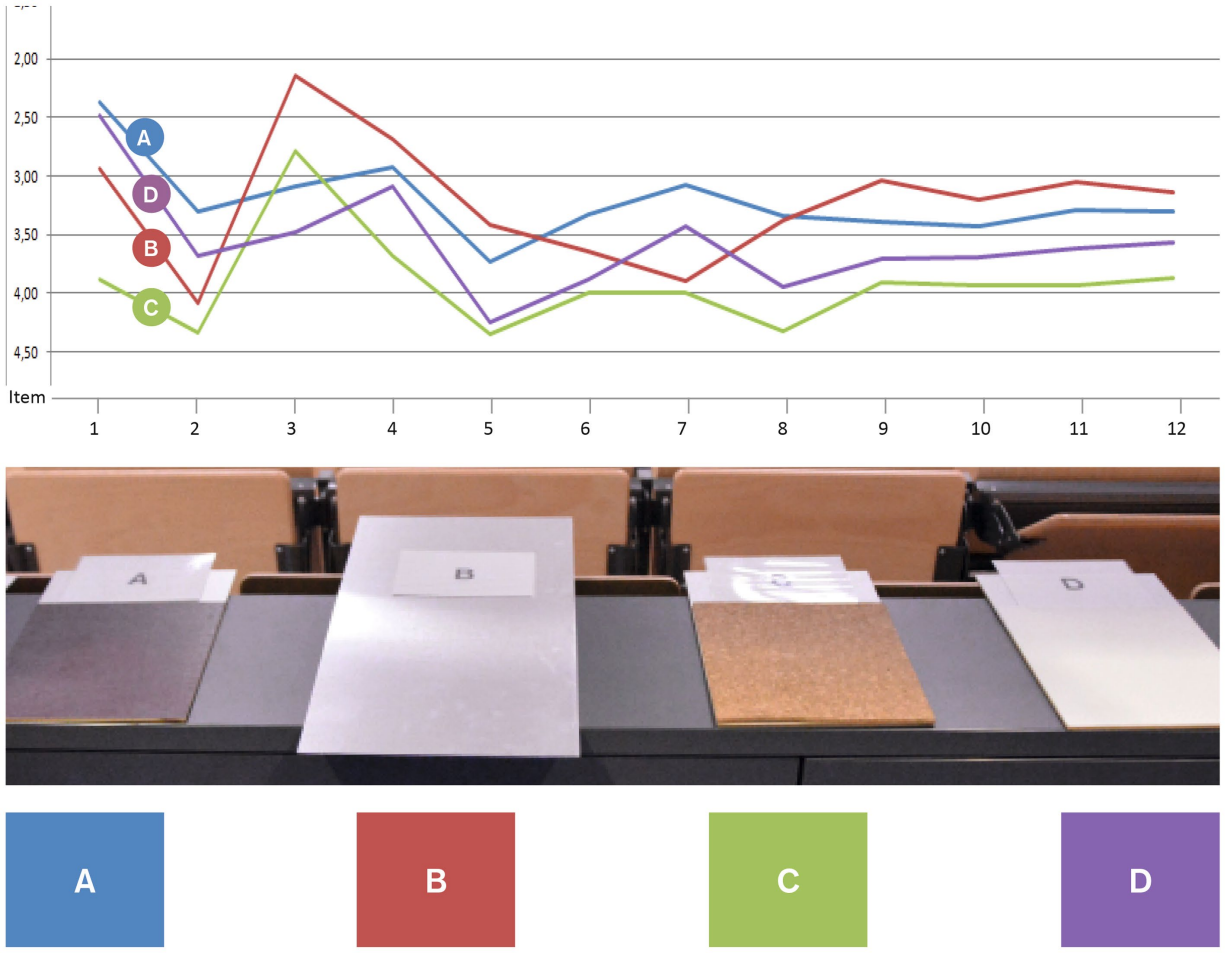
Design feature “haptic sensation while writing.”

These explorative designs formed the basis of another empirical pre-test focussing on the notion of embodiment while interacting with the lectern. Therefore, the four formal-aesthetic parameters (1) basic shape, (2) handle form, (2) material and colour of the lectern and (4) haptic sensation while writing were investigated (Figures 3–7). The pre-test was conducted on 2 days during a lecture at Darmstadt University of Applied Sciences in January 2015. The sample therefore comprised students between the ages of 18 and 36. Since participants could voluntarily decide how many stations they wanted to participate in, station sample sizes varied between 62 and 76 per station (*n*_1_ = 75, *n*_2_ = 62, *n*_3_ = 66, *n*_4_ = 76), of which 24–29 were female and 31–46 were male participants. Participants evaluated all four parameters using a semantic differential based on 12 item-pairs on a 7-point-scale (e.g., “When writing on the writing pads A, B, C and D, I feel…” coded from “relaxed” to “nervous”).

#### 2.3.1 Basic shape

The parameter “basic shape” refers to the overall shape of the table top of the lectern. Four versions were designed and produced from wood (Figure 4). These shapes were designed based on the assumption that a typical body posture standing at the lectern would be to hold onto the edged left and right of the table top. Therefore, different angles and widths of this part would stimulate different postures of the upper body, namely a more open vs. close posture. With regards to evidence from embodiment research (e.g., Tschacher and Storch, 2012), we hypothesised that a more open posture would increase users’ sense of self-efficacy, confidence and control. In this category of design features, shape A (a parallel design with a width of 60 cm) scored highest with regards to the targeted qualities perceived by users (i.e., feeling supported, motivated, empowered) (see Figure 4) and was therefore selected as a building block for the design of the positive prime.

#### 2.3.2 Handle shape

Following the assumptions regarding the basic shape, we assumed furthermore that the shape of the handles – i.e. the volume and formal characteristics of the edges – has an additional effect on the aforementioned mental constructs. Therefore, four versions of this handle were designed with varying degrees of volume and other formal detail (see Figure 5). Handle design B scored highest with regards to the targeted effects perceived by users (i.e., feeling supported, motivated, empowered) in this category of design features and was thus selected as another building block for the design of the positive prime.

#### 2.3.3 Material and colour

A growing body of evidence illustrates the profound effects material and colour can have on individuals (e.g., Tsunetsugu et al., 2007; Mehta and Zhu, 2009; Lobel, 2014). Analogously to the aforementioned mood boards, we designed three material combinations each representing a typical furniture style, (A) “clean and reduced,” (B) “fresh and friendly” and (C) “heavy and strong.” Participants were invited to touch the material boards in order to bodily experience the material characteristic. Combination C (dark wood and leather) scored highest with regards to the targeted effects perceived by users (i.e., feeling supported, motivated, empowered) in this category (see Figure 6) and was thus selected as a third element for the design of the positive prime.

#### 2.3.4 Haptic sensation while writing

The most intense interaction with the lectern was supposed to occur when participants would write on the table top in order to evaluate the product they were asked to test. Therefore, the fourth station comprised four different materials to write onto, (A) hard leather, (B) metal, (C) cork, (D) soft leather (see Figure 7). Overall, hard leather scored highest in this category with regards to the targeted effects perceived by users (i.e., feeling supported, motivated, empowered) due to its best writing usability (i.e., scored highest for the items “Writing pads A, B, C and D are when writing on them …” coded with “comfortable” vs. “uncomfortable”) and was thus selected as a design element of the positive prime.

### 2.4 Subsequent iterative design process

**Figure 8:**
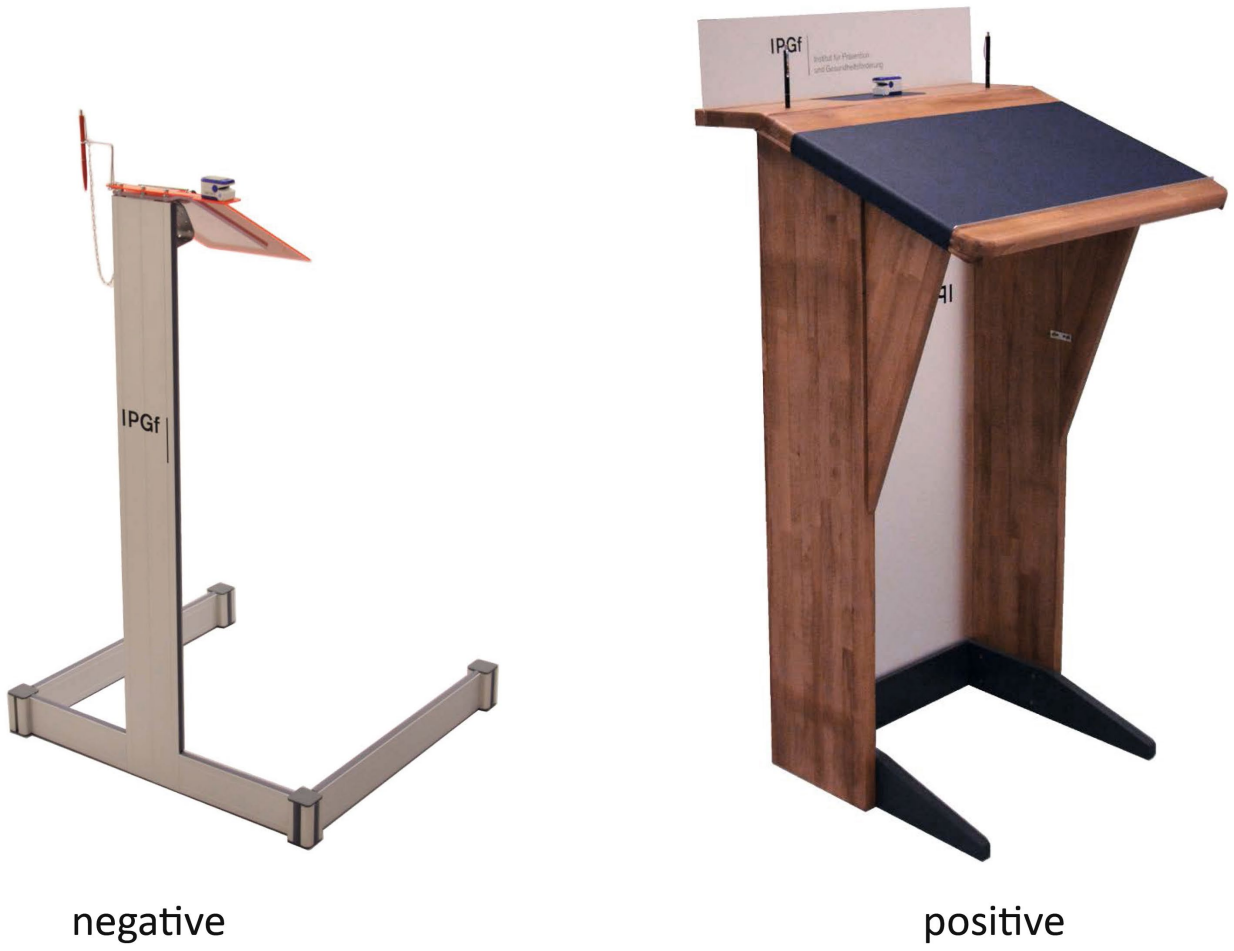
Final prototypes of “positive” and “negative” design primes.

Based on these pre-tests, an iterative design process continued that comprised several design concepts developed as scetches and CAD models before being built as miniature models and eventually manufactured as real size prototypes from (A) wood, hard leather and acrylite or (B) alumium profiles and acrylite (see Figure 8). The “positive” design prime resembles a robust and massive wooden lectern providing excessive space for writing on top of it. Its material properties, comfort and overall aesthetic appeal are chosen to represent valorisation for the task and the participant. The “negative” design prime is based on a rather functionalist, clean and technical setup made from alumium profiles and a red top part. Its writing surface is almost too small to write comfortably on it and slightly gives way when pressure is applied, which is supposed to emphasise qualities of weakness and uncertainty. The colour red was chosen to additionally exacerbate cognitive task solving (Mehta and Zhu, 2009). Adding to the clinical and sterile aesthetics of the overall design, the legs of the lecture build a squarelike shape embracing the participant and by this aiming to limit their subjectively experienced freedom of movement while using the lectern. All in all, in contrast to the “positive” design prime, most of the formal-aesthetic features of this design communicate less valorisation and hinder users’ performance potentially compromising their situational sense of competence and self-efficacy.

### 2.5 Evaluation of design primes and manipulation check

**Figure 9:**
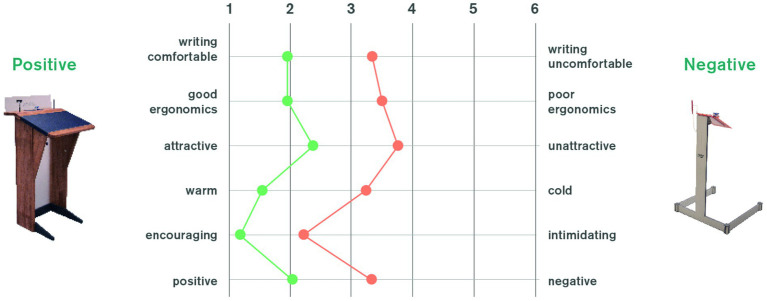
Semantic differential to evaluate both design primes with regards to intended perception.

To confirm whether participants actually perceived the design prime the way they were intended to, participants were asked at the end of the actual randomised control trial (see 3.2) to rate the lectern they had used on a 6-item semantic differential. As expected, the positive design scored significantly higher on all items confirming its overall design “style” as being more encouraging, usable and attractive (see Figure 9).

## 3 Methods B: testing of design primes

### 3.1 Participants

To test the hypothesised effects of the designed primes on mental concepts of the people using them, an experiment was administered. The experiment took place independently in two different locations, (a) a University in Darmstadt, Germany, and (b) a Clinic for orthopaedic rehabilitation in Bad Kreuznach, Germany. In the University setting, *n* = 83 students (35 male, 48 female; median age between 22 and 25) took part in the study. In the clinical setting, *n* = 38 patients of the orthopaedic rehabilitation clinic took part (12 male, 25 female, 1 unknown); median age between 51 and 60 years). In both settings, participants were allocated randomly to one of two groups that were presented with the two lecterns described above. Group 1 (university: *n* = 41; clinic: *n* = 19) was presented with the stable, aesthetically favourable lectern (“positive condition,” see Figure 8, left). Group 2 (university: *n* = 42; clinic: *n* = 19) was presented with the unstable, aesthetically less favourable lectern (“negative condition,” see Figure 8, right).

### 3.2 Procedure

**Figure 10:**
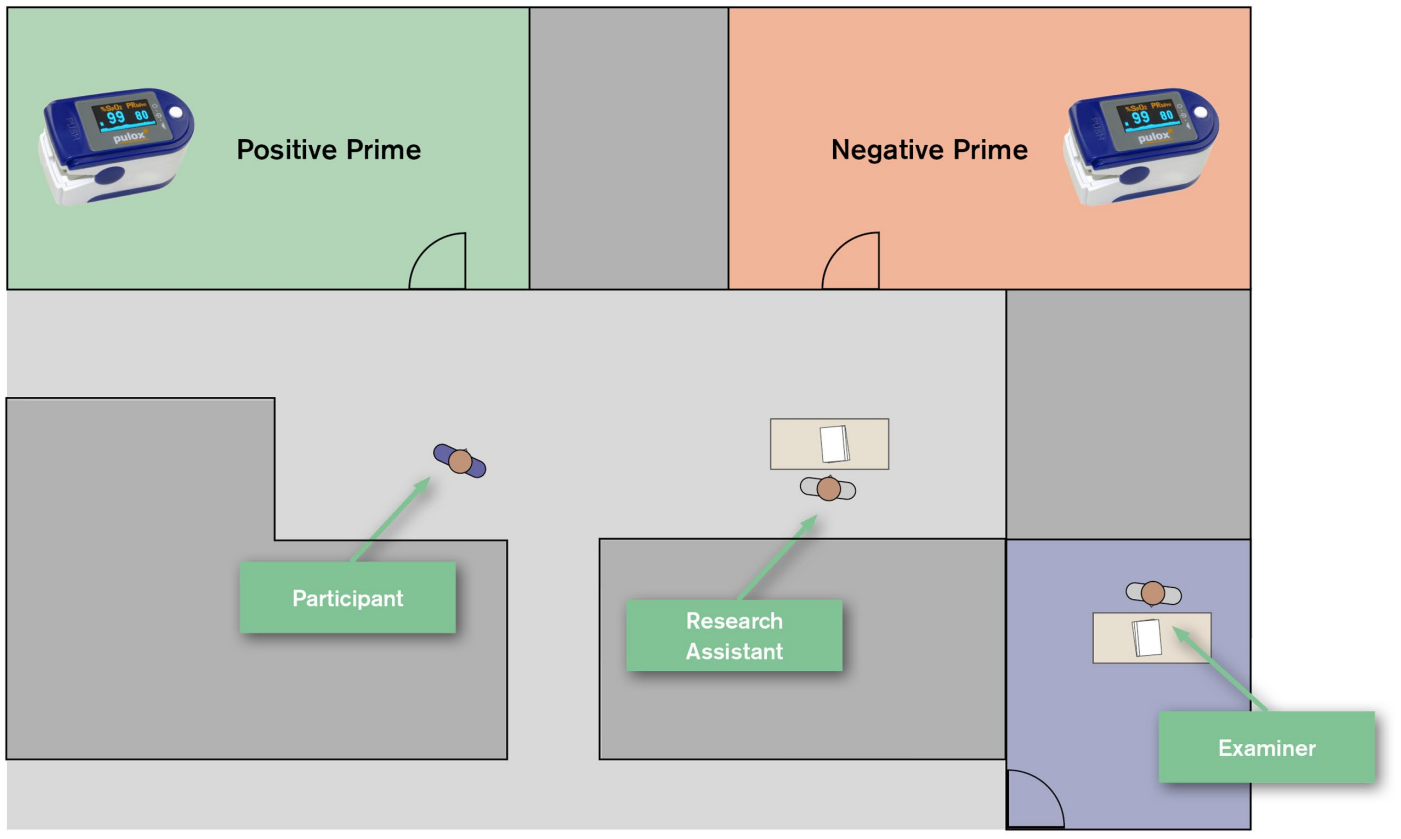
Schematic illustration of study design as randomised control trail.

To test the effect of both primes, participants were asked to evaluate an unrelated medical product (a pulse oximeter). Participants were recruited via advertisements, e.g., on posters, a week prior to the experiment. On the day of the experiment, participants approached the research assistant, received an envelope and were randomly assigned to one of two rooms (positive vs. negative condition). The actual location of the primes was alternated after each day, decreasing the likelihood of other environmental factors in the room to potentially affect the outcomes. The research assistant assigned participants alternating between both rooms or depending on which room was free. For each room, a set of papers was prepared in an enveloped label with a code relating to one of the two primes that was only known to the examiner. The assistant was unaware of which prime was placed in which room and of the actual goal of the experiment (double blind condition). In the room, the pulse oximeter was placed on the lectern while the lectern was the only useful piece of furniture to write on. After filling out the questionnaire, participants were asked to put the papers back into the envelope and go to the examiner in a different room to collect their incentives. Once participants had handed in the envelope, the actual background of the study was revealed to them behind closed doors (see Figure 10).

**Figure 11:**
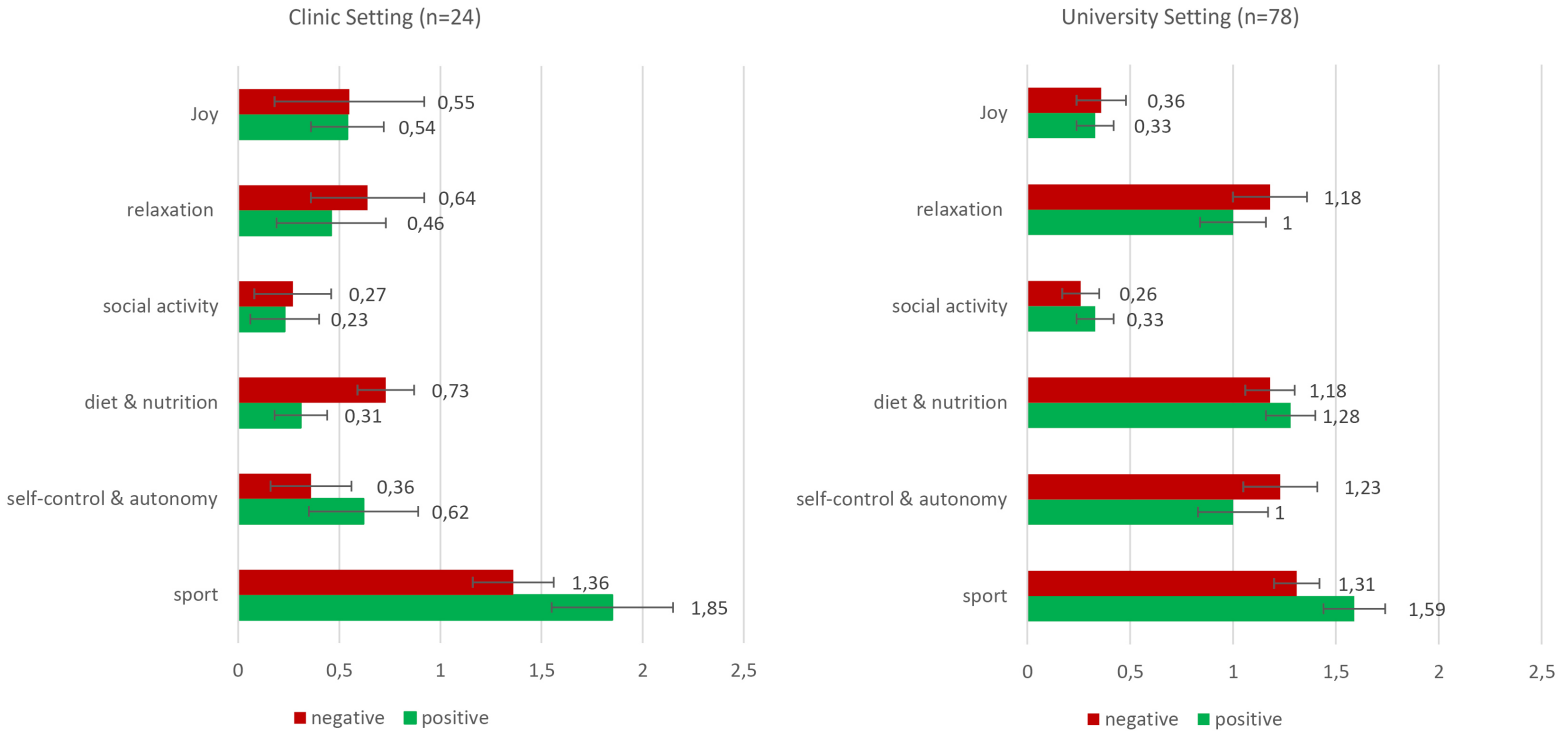
Absolute mentioning frequencies in clinical setting and university setting (means and standard errors).

### 3.3 Instruments

To evaluate the effect of the design primes a six pages questionnaire was developed comprising 46 items and an open-ended mentioning task. The first six items referred to the product participants thought to evaluate (e.g., “The product is easy to use.”). Furthermore, the questionnaire included a 10-item scale on general self-efficacy (WIRKALL_r; Schwarzer and Jerusalem, 1999), an 11-item scale on resiliency (RS11, Schumacher et al., 2005), a 9-item scale on sense of coherence (SOC-L9; Schumacher et al., 2000) and a 4-item short scale on sense of control (KÜ4, Kovaleva et al., 2012). Participants rated their agreement on a 4-point Likert-type scale ranging from 1 = “not true” to 5 = “totally true” (WIRKALL_r; α = 0.89), on a 7-point scale with varying codings (e.g., “very often” to “hardly or never”) (SOC-L9; α = 0.85), on a 7-point scale ranging from 1 = “do not agree” to 7 = “fully agree” (RS11; α = 0.88) and on a 4-point Likert-type scale ranging from 1 = “does not apply” to 5 = “fully applies” (KÜ4; α = 0.74). For this article, the most relevant element was a mentioning task at the end of the questionnaire asking participants to list all health-promoting activities for daily life that come to their mind. In addition to that, they were asked to indicate which of these activities they (a.) are already doing, (b.) assume they could do or (c.) do not believe to be able to do. Apart from these items, sociodemographic items were included (i.e., age, gender).

### 3.4 Data analyses

The items mentioned in the mentioning task were clustered into six categories derived from the data (diet & nutrition, sport, social activity, relaxation, joy, self-control & autonomy). The absolute mentioning frequency comprises all aspects independently from whether participants commented that they could implement them or not. In the clinical setting, from each group, several participants did not provide data on this task (group 1: *n* = 6; group 2: *n* = 8) and were thus excluded from the analysis. Similarly, in the university setting, *n* = 5 (group 3: *n* = 2; group 4: *n* = 3) participants did not provide data on this task and were excluded. Remaining sample sizes are *n* = 13 (group 1), *n* = 11 (group 2), *n* = 39 (group 3) and *n* = 39 (group 4). As hypothesised earlier, mentioning frequency of words that are related to physical activity pose the central variable of interest.

Group differences were assessed for two variables regarding mentioning frequency: absolute mentioning frequency and relative mentioning frequency. To account for verbal fluency, absolute mentioning frequencies of aspects related to physical activity were tallied and standardised by the overall sum of health-promoting activities mentioned. Resulting scores range from 0 to 1, with higher scores indicating stronger focus on aspects related to physical activity in all mentions. Before examining group differences in mentioning frequency of physical activity aspects, for the clinical setting, a Kolmogorov–Smirnov test indicated normal distribution of the relative mentioning frequency variable (*p* > 0.05) but not of the absolute mentioning frequency variable (*p* < 0.001). For the university setting, a Kolmogorov–Smirnov test indicated no normal distribution of the relative mentioning frequency variable and of the absolute mentioning frequency variable (*p* < 0.001). Hence, group differences in variables without normal distribution were assessed with a Mann–Whitney-U test as a non-parametric alternative to the *t*-test.

## 4 Results

Overall, t-tests on all four standardised scales did not reveal significant differences regarding general self-efficacy, resiliency, sense of coherence or sense of control between negative and positive condition both in the university and clinic setting, all *p*s > 0.05.

In both settings, first, absolute mentioning frequencies of physical activity related aspects were calculated (clinical group 1: *M* = 1.85; SD = 1.07; clinical group 2: *M* = 1.36; SD = 0.67; university group 3: *M* = 1.59; SD = 0.91; university group 4: M = 1.31; SD = 0.69; for all mentioning frequencies see Figure 11). In the university setting, a Mann–Whitney-U test revealed no significance *U* = 633.50, *p* = 0.14, *r* = 0.17. In the clinical setting, a Mann–Whitney-U test revealed no significance, yet a close to medium effect size *sensu* Cohen (1988), *U* = 50.00, *p* = 0.23, *r* = 0.28.

For relative mentioning frequencies in the clinical group (clinical group 1: *M* = 0.51; SD = 0.31; clinical group 2: *M* = 0.43; SD = 0.26), an Independent Samples t-test showed no significance, *t* [22] = 0.66, *p* = 0.52, *d* = 0.27. Here again, effects are of close to medium size. In the university group (university group 1: *M* = 0.32; *SD* = 0.18; university group 2: *M* = 0.26; SD = 0.13), a Mann–Whitney-U test showed no significance *U* = 618.00, *p* = 0.15, *r* = 0.16. Due to highly differing sample sizes, no overall group comparison was performed.

## 5 Discussion, limitations and conclusion

This paper presented the structured and evidence-based development and application of two lecterns as design primes to influence people’s self-efficacy, sense of control, sense of coherence and resiliency after using them as well as an activation of thoughts on health-promoting activities. These assumptions were tested in a university setting and in a clinical setting. This process of developing health-promoting design primes and the process of systematically evaluating their effectiveness are the key outputs of this paper.

With regards to the actual empirical results of the priming experiment, we did not find any effect on the psychological constructs of self-efficacy, sense of control, sense of coherence and resiliency. However, clinical participants using the positive design prime showed stronger tendencies to mention more health-promoting activities related to physical aspects than participants using the negative one did. In a clinical setting patients, staff and carers are constantly confronted with a high number of formal-aesthetic stimuli that might trigger associations that otherwise – often subtly – affect their emotional and cognitive states. Purposely designing at least some of these design features applying evidence-based design principles offers new and potentially psychosocially supportive approaches for healthcare design and design research. These results provide (a.) additional evidence for the overall effect mechanism of built environment design primes on mental concepts and (b.) new insights on pathology-related effects of design primes, i.e., an effect on a specific pathology-related domain. This points to the direction making use of design primes in clinical contexts to address and foster health-related behaviours through design.

From a design research point of view, this paper illustrated how an iterative approach can provide adequate, evidence-based design primes, which can be a fruitful source for other studies in the field. Small sample sizes limit the ecological validity of the current study, as they are prone to outliers, which increases type 1 error. Thus, the results presented here require careful interpretation and do not claim generalizability. Especially as there were no baseline assessments, intergroup differences before the prime intervention are possible. Further studies using bigger and more balanced samples and a non-clinical young-old adult group as reference would allow to extent and enrich the current findings.

**Figure 12:**
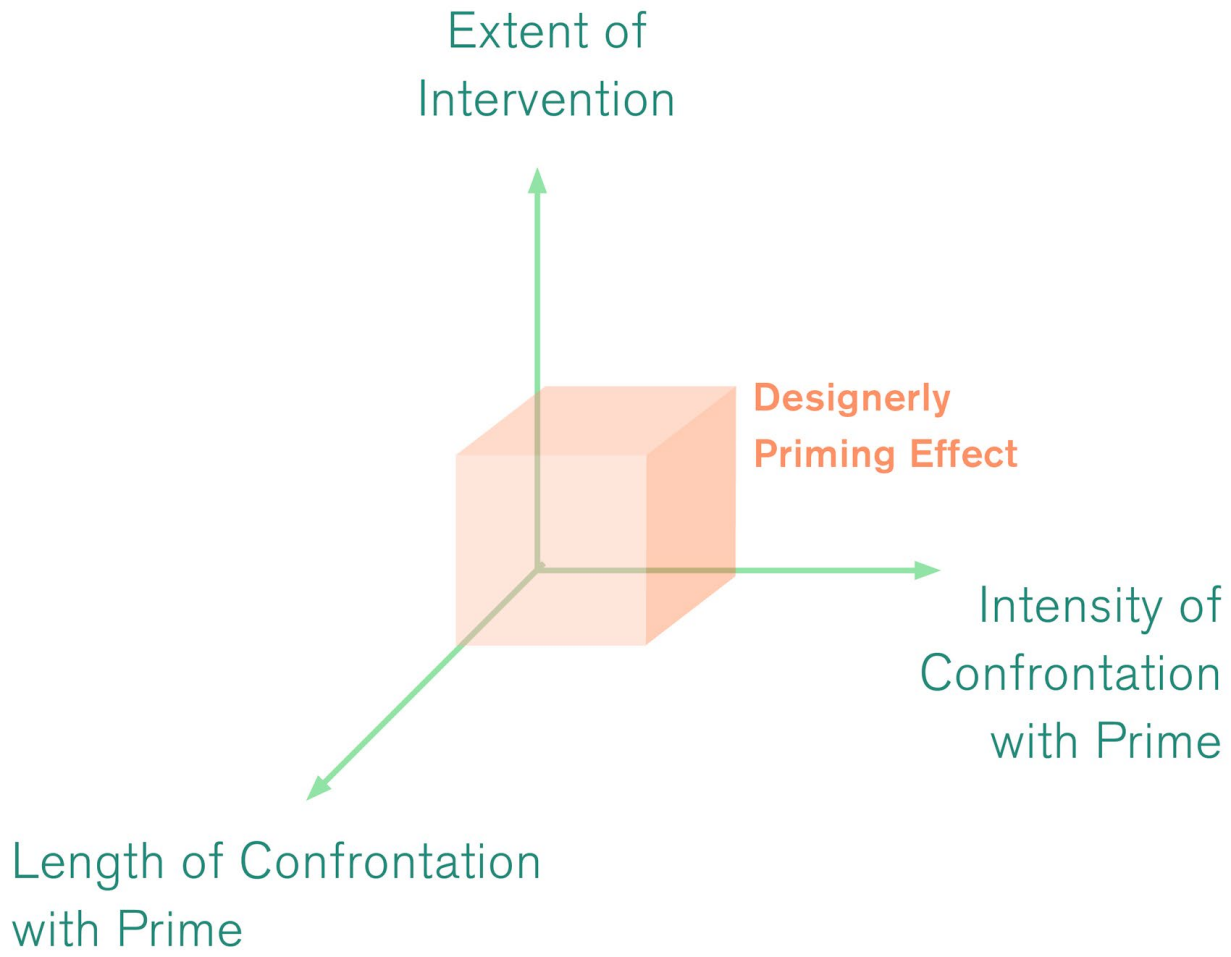
Potential ways of reinforcing the effect.

From these findings, we identify three potential ways of reinforcing the intended effect (see Figure 12): Participants in the experiment presented in this paper interacted with the primes only for a rather short period of time (*ca.* 2–8 min). In other contexts, users might interact with a product for much longer durations (e.g., during a therapy or training session or while being in bed). Second, participants in this experiment only indirectly interacted with the primes by writing on them. More intense confrontation with design primes (e.g., by holding, moving or using them) might reinforce its effect further. Thirdly, the design primes used in this experiment were limited to one piece of furniture while the overall context remained the same. As a next step, we would propose a more complex design intervention including, e.g., the entire interior design (flooring, walls, lighting, other pieces of furniture) as that might create synergies and thus further increase the intended effect. This more holistic approach is typical for priming approaches in the retail context. Accordingly, although we only found small effects, we have sufficient reason to assume that the aforementioned formal-aesthetic design features might influence thoughts, emotions and behaviours with regards to health behaviour and more. We further argue that in clinical contexts these effects can be reinforced or modulated by the pathologies of the patients that interact with these primes and are thus pathology-related. The research translated an established practice in retail, i.e., priming, to a health context and demonstrated that there is potential for priming to be used for health promotion through therapeutic design interventions. This remains an important task, as design primes promoting or motivating health-behaviour might be powerful and cost-efficient assets to public health.

All in all, this paper primarily emphasises the role and responsibility of design interventions in therapeutic built environments. It supports the current tendency of evidence-based design decisions and expands this approach to the complex field of formal-aesthetic design. Therefore, design primes as physical artefacts presented here need to be seen as exploratory probes to investigate the potentials formal-aesthetic feature can have. More research is needed to understand and operationalise these manifold effects that formal-aesthetic features such as colour, haptics, scent, etc. can have on patients and other vulnerable user groups. Special attention should be paid to the fact that specific aspects such as pathology-related symptoms might affect the working mechanism of design primes and thus influence the way formal-aesthetic design features can support or otherwise impede therapeutic processes. While we argue the underestimated role formal-aesthetic design can have in therapeutic settings, it is merely one dimension of the complex field of healthcare design. Only if other aspects such as pragmatic functionality, service processes, hygiene and many others are included, this approach can exert its full potential. Furthermore, formal-aesthetic design features are no separate category that works on its own but are strongly intertwined with the aforementioned aspects. The entirety of all perceivable features of a setting will create a specific experience, which affects the therapeutic outcomes one way or the other.

## Data availability statement

The raw data supporting the conclusions of this article will be made available by the authors, on legitimate request by scholars.

## Ethics statement

Ethical approval was obtained internally by supervising scholars. The studies were conducted in accordance with the local legislation and institutional requirements. The participants provided their written informed consent to participate in this study.

## Author contributions

All phases of the empirical research including pre-tests were conducted by JR-G and supervised by KS. The primes and all other physical artefacts were designed and built by JR-G. The statistic evaluations of the final experimental research were conducted and evaluated by HM. The conceptual analysis and theoretical conclusion were collaboratively developed by all authors. All authors contributed to the article and approved the submitted version.
